# Recent update on barbiturate in relation to brain disorder

**DOI:** 10.17179/excli2021-3687

**Published:** 2021-06-07

**Authors:** Sachchidanand Pathak, Gaurav Gupta, Lakshmi Thangavelu, Sachin K. Singh, Kamal Dua, Dinesh Kumar Chellappan, Ritu M. Gilhotra

**Affiliations:** 1 School of Pharmacy, Suresh Gyan Vihar University, Mahal Road-302017, Jagatpura, Jaipur, India; 2 Department of Pharmacology, Saveetha Dental College and Hospitals, Saveetha Institute of Medical and Technical Sciences, Saveetha University, Chennai, India; 3 School of Pharmaceutical Sciences, Lovely Professional University, Phagwara, Punjab, 144411, India; 4 Discipline of Pharmacy, Graduate School of Health, University of Technology Sydney, NSW 2007, Australia; 5 Department of Life Sciences, School of Pharmacy, International Medical University, Bukit Jalil 57000, Kuala Lumpur, Malaysia

## ⁯

***Dear Editor, ***

Barbiturate is a potent substance which forms a quintessential part of the NDPS Act. The substance is categorized under the psychoactive groups of drugs and is essentially a drug that possesses both hypnotic and sedative properties. The precursor for barbiturate is barbituric acid which is a condensation product of malonic acid and urea. However, barbituric acid itself is not a centrally acting depressant. Diethylbarbituric acid (Veronal) is the first ever barbiturate with hypnotic properties that was used as early as 1903 (Hadjihambi et al., 2020[5]). The drug induced sleep both in human and animals. The substance was also called as barbital. Later in the year 1912, a second barbiturate drug, phenobarbitone was introduced into clinical practice which had both sedative and hypnotic properties. The phenomenal success of both these drugs announced the beginning of the barbiturate era. Their influence as the pre-eminent sedative-hypnotic agents was felt for over half a century. Although several so-called non-barbiturate drugs attempted to displace the barbiturates from their pinnacle from time to time, it was not until 1961 when a substance named chlordiazepoxide was introduced into the market that their position was seriously challenged (Velle et al., 2021[23]). Several earlier studies have reported the characteristic features and the severity of the barbiturate withdrawal syndrome. In cases of mild withdrawal syndrome, symptoms like apprehension, hyperexcitability, mild tremors, loss of appetite and piloerection were observed. An intermediate withdrawal syndrome exhibited tightness in the muscles, extreme tremors, sudden loss of body weight, altered motor activity, excessive nausea, and vomiting (Sharpe et al., 2020[16]). The hallmarks of a severe withdrawal syndrome are convulsions, delirium or hallucination and hyperthermia or unusually high fever. The severity of withdrawal syndrome has been shown to depend on the frequency of drug administration and the duration of action of the drug. We review recent research on the role of barbiturates in brain disorders in this letter (Table 1(Tab. 1); References in Table 1: Brandt et al., 2018[1]; Chakraborty and Hocker, 2019[2]; Colton et al., 2014[3]; Forsyth et al., 2003[4]; Hadjihambi et al., 2020[5]; Hocker et al., 2018[6]; Klein et al., 2015[7]; Lewis and Adams, 2021[8]; Mairinger et al., 2012[9]; Mansour et al., 2013[10]; Murphy et al., 2020[11]; Ryu et al., 2019[12]; Sakuma et al., 2020[13]; Sánchez Fernández et al., 2019[14]; Schizodimos et al., 2020[15]; Shein et al., 2016[17]; Specchio and Pietrafusa, 2020[18]; Tat et al., 2017[19]; Töllner et al., 2014[20]; Tremont-Lukats et al., 2008[21]; Velle et al., 2019[22]; Wang et al., 2018[24]; Xie et al., 2009[25]; Young et al., 2016[26]; Zhang et al., 2019[27]).

## Conflict of interest

The authors declare no conflict of interest.

## Figures and Tables

**Table 1:**
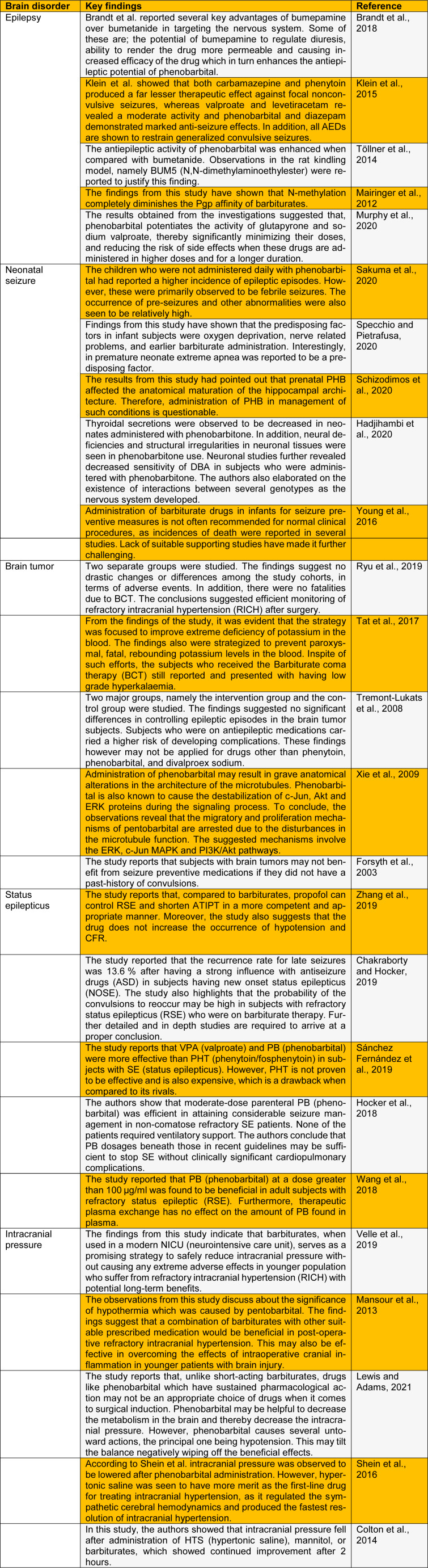
Recent study on the role of barbiturates in brain disorders
